# Anti-PD-1 immunotherapy combined with stereotactic body radiation therapy and GM-CSF for the treatment of advanced malignant PEComa: A case report

**DOI:** 10.3389/fonc.2023.1045119

**Published:** 2023-04-18

**Authors:** Yunfan Wang, Weiling Li, Xin Zuo, Ke Min, Yuehua Tang, Hong Chen, Weimin Wang, Yan Zhou

**Affiliations:** ^1^ 1Department of Oncology, The Affiliated Yixing Clinical School of Medical School of Yangzhou University, Yangzhou University, Yixing, Jiangsu, China; ^2^ Obstetrics and Gynecology, The Affiliated Yixing Clinical School of Medical School of Yangzhou University, Yangzhou University, Yixing, Jiangsu, China

**Keywords:** PEComa, immunotherapy, radiotherapy, PD-L1, GM-CSF

## Abstract

**Background:**

Perivascular epithelioid cell neoplasm (PEComa) is a rare mesenchymal tumour. Due to its low incidence, a standard treatment regimen for PEComa has not yet been established. Radiotherapy has a synergistic effect with PD-1 inhibitors and GM-CSF. We treated advanced malignant PEComa with a triple regimen of PD-1 inhibitor, SBRT and GM-CSF to provide better therapeutic effect.

**Case presentation:**

A 63-year-old woman was diagnosed with malignant PEComa after presenting with postmenopausal vaginal bleeding. Despite two surgeries, the neoplasm eventually metastasized throughout the body. We formulated triple therapy with SBRT, a PD-1 inhibitor, and GM-CSF for the patient. The patient’s local symptoms were controlled at the radiotherapy site, and the lesions at the unirradiated sites were also relieved.

**Conclusions:**

For the first time, a triple regimen of PD-1 inhibitor, SBRT and GM-CSF was used in the treatment of malignant PEComa and achieved good efficacy. Considering the lack of prospective clinical studies in PEComa, we believe that this triple therapy is a good-quality regimen for advanced malignant PEComa.

## Introduction

Perivascular epithelioid cell neoplasm (PEComa) is a rare mesenchymal tumour. PEComa was defined by the World Health Organization (WHO) as a “mesenchymal tumour composed of histologically and immunohistochemically distinct perivascular epithelioid cells”. It includes angiomyolipoma (AML), clear cell sugar tumours (CCSTs), lymphangioleiomyomatosis (LAM), Clear-cell myomelanocytic tumours(CCMTs), and other unusual clear cell tumours (1). PEComas have different biological behaviours, and approximately one-third are malignant PEComas (2). The cause of PEComa is unknown, but more than 25% of them occur in the uterus or cervix (1). PEComa tumour cells have the unique morphological feature of “perivascular epithelioid cells”. In immunohistochemistry, the tumour expressed both melanocyte markers (HMB45, Melan-A) and smooth muscle markers (SMA, desmin). Among them, HMB45 is the most characteristic. It is positive in almost 100% of PEComas (3).

Surgical resection with negative margins is the most important treatment for PEComa (4). The role of radiation therapy and programmed cell death protein 1 (PD-1) inhibitors is currently unclear. It has only been explored in a few patients (5, 6). Recently, mammalian target of rapamycin (mTOR) inhibitors were found to be potentially effective in some advanced PEComas. In November 2021, albumin-bound sirolimus was approved by the U.S. Food and Drug Administration (FDA) for advanced malignant PEComa. This drug had an overall response rate of 39% in clinical trials (7). However, many patients cannot use it due to a variety of factors, including price and drug availability.

In recent years, immunotherapy represented by PD-1/programmed cell death 1 ligand 1 (PD-L1) inhibitors has profoundly changed the treatment of malignant tumours. However, there are large differences in the response of different tumours to PD-1 inhibitors, which may be related to tumour PD-L1 expression (8). Regrettably, due to the low incidence, the expression of PD-L1 in PEComa has not been monitored by the research team to date. Many studies have shown that PD-1 inhibitors in combination with radiation therapy or Granulocyte-macrophage Colony Stimulating Factor (GM-CSF) can enhance antitumor effects. This may be related to the activation of the cGas-STING pathway, the release of tumour-associated antigens (TAAs), and changes in the immune microenvironment within the tumour (9–11). Studies have shown that multisite stereotactic body radiotherapy (SBRT) combined with PD-1 inhibitors may achieve better clinical outcomes (12, 13). The triple use of PD-1 inhibitors, GM-CSF and SBRT may be more likely to produce abscopal effects (14, 15).

Here, we report a case of advanced malignant PEComa. The patient underwent 2 surgeries, but the neoplasm eventually progressed systemically. After we administered triple therapy with anti-PD-1, GM-CSF and SBRT, the local symptoms of the patient were significantly relieved, and distant metastasis was effectively controlled.

## Case presentation

**Figure 1:**
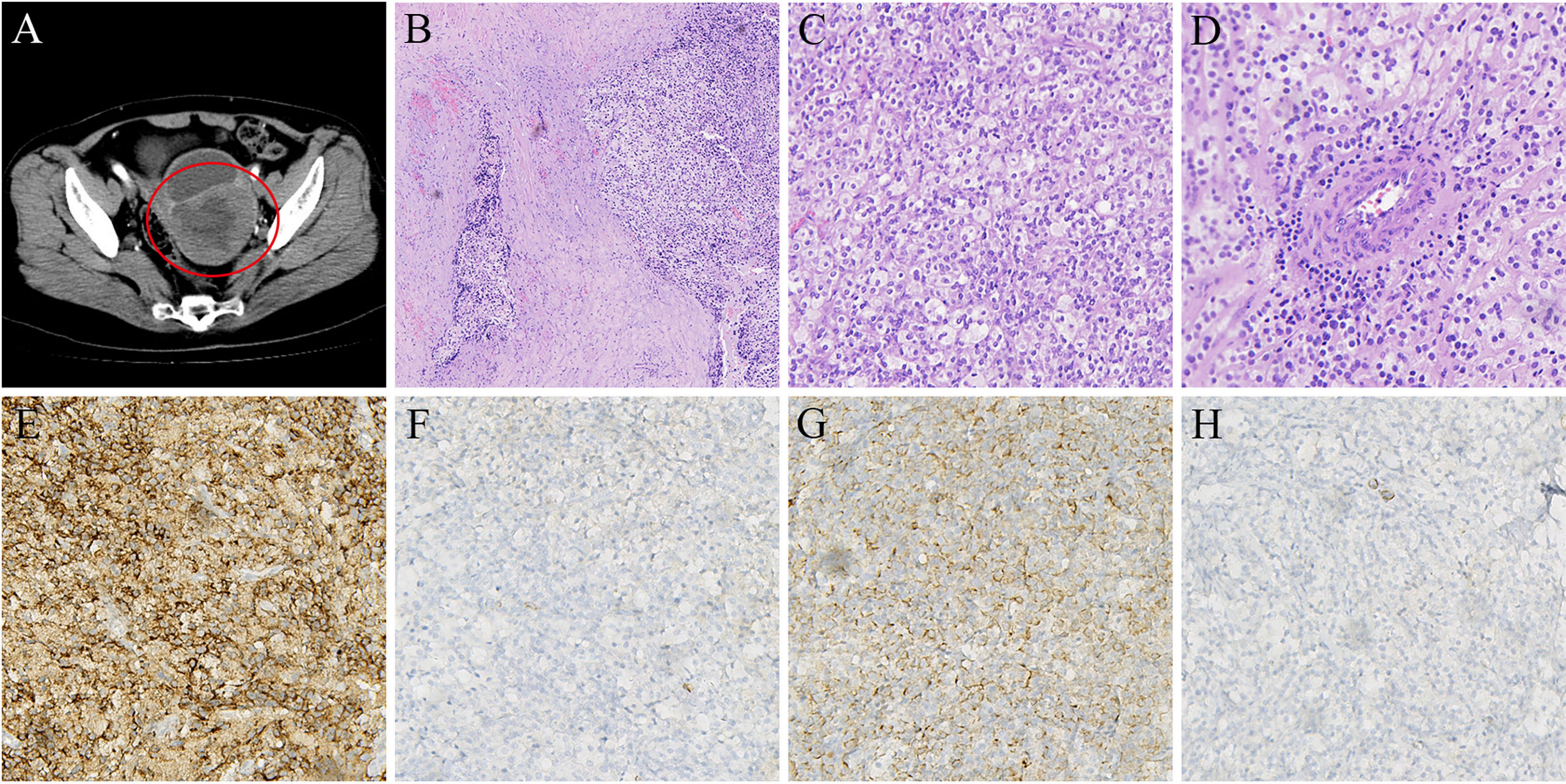
**(A)** CT scan shows primary mass in posterior wall of uterus (in 2017). **(B)** HE staining indicated that tumor cells showed invasive growth (5×). **(C)** Tumor cells are epithelioid cells with clear or eosinophilic cytoplasm (20×). **(D)** Tumor cells are densely distributed around blood vessels(20×). **(E)** Immunohistochemical stain positive for HMB-45 (20×). **(F)** Immunohistochemical stain negative for Melan-A (20×). **(G)** Immunohistochemical stain weakly positive for Desmin(20×). **(H)** Immunohistochemical stain negative for SMA (20×).

In 2017, a 63-year-old woman with no underlying medical conditions was hospitalized with postmenopausal vaginal bleeding. A CT scan showed a cystic and solid mass in the posterior wall of the uterus (**Figure 1A**), suggesting the possibility of malignancy. On the advice of a gynaecologist, she underwent a total hysterectomy and double adnexectomy. Gross pathology showed that the uterine cavity was filled with a cauliflower-like tumour measuring approximately 10 cm × 10 cm. The cut surface was grey and white with necrosis. The mass invaded the full thickness of the uterine muscle wall but did not break through the serosal layer, and the surgical margins were negative. The pelvic and common iliac arterial lymph nodes were not invaded. Histological sections showed aggressive tumour growth (**Figure 1B**). Tumour cells were epithelioid cells with clear or eosinophilic cytoplasm (**Figure 1C**), and they tended to aggregate around blood vessels (**Figure 1D**). Immunohistochemical staining showed that the melanocyte marker HMB45 was positive, and Melan-A was negative (**Figures 1E, F**); the smooth muscle marker desmin was weakly positive, and SMA was negative (**Figures 1G, H**). In addition, ER and vimentin were positive, and S-100, PR, Syn, HER-2, actin, CD10, and CD56 were all negative. Due to risk factors such as tumour size larger than 5 cm, aggressive growth, and necrosis, the lesion was classified as a malignant PEComa according to the classification of Folpe et al. (1).

**Figure 2:**
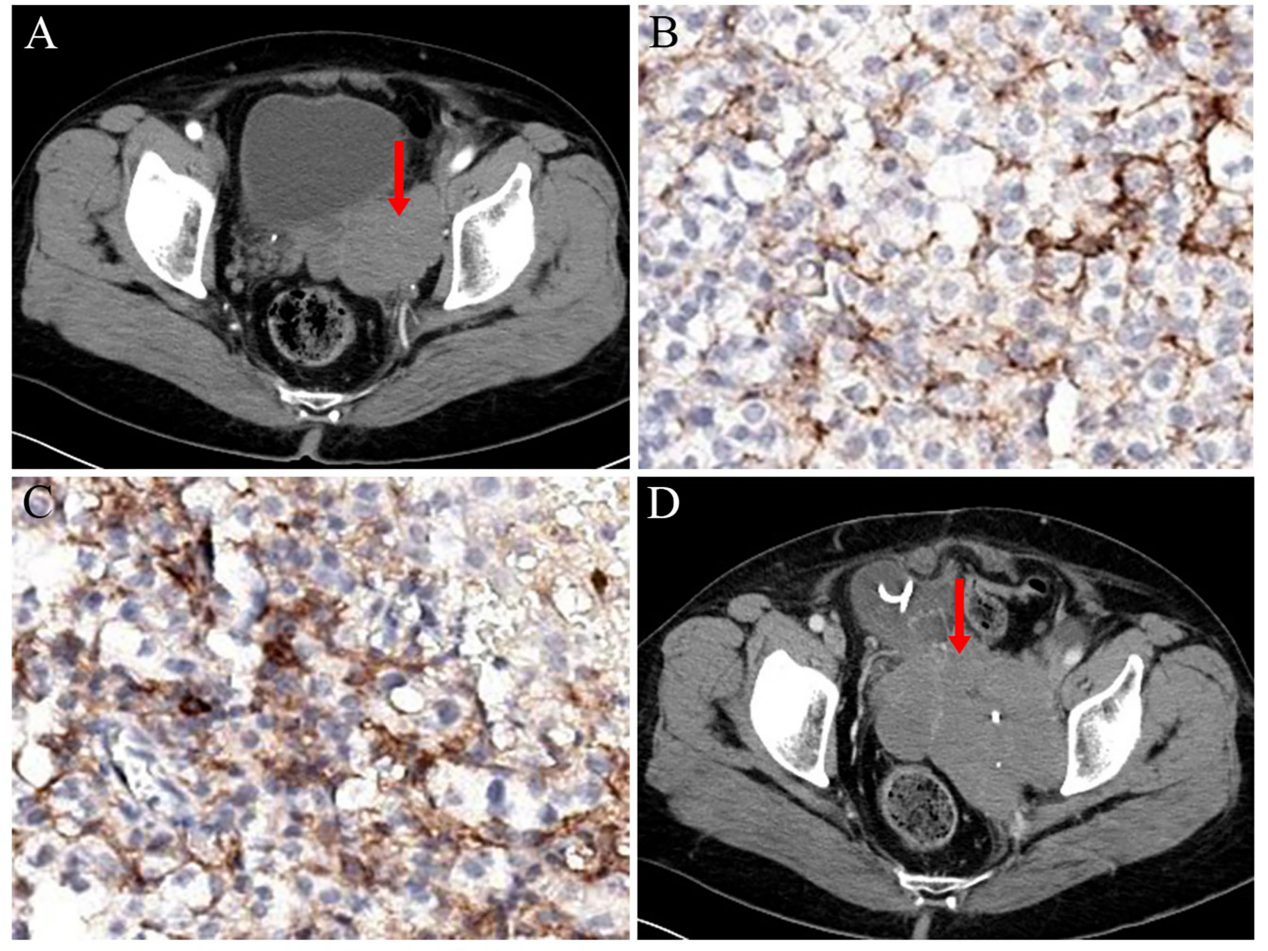
**(A)** CT scan showed a pelvic space-occupying lesion (in 2020). **(B)** TPS of tumor PD-L1 expression: Number of PD-L1 stained tumor cells/total tumor cells (PD-L1 stained and non-stained) ×100%. **(C)** CPS of tumor PD-L1 expression: Number of PD-L1 stained cells (tumor cells, lymphocytes, macrophages)/total tumor cells (PD-L1 stained and non-stained) ×100. **(D)** CT scan revealed another large mass in the pelvis (in 2022).

For the next 3 years, the patient’s condition was stable. Then, in September 2020, when the patient was re-examined with CT, a tumour of approximately 8 cm × 4 cm was found in the pelvis (**Figure 2A**). Considering the high possibility of recurrence, the patient underwent pelvic tumour resection and left ureteral double J-tube placement again. Postoperative pathological diagnosis was still malignant PEComa. The detection of PD-L1 in the tumour showed a tumour proportion score (TPS) of 2% and combined positive score (CPS) of 5 (**Figures 2B, C**). However, the patient was not on antitumor therapy. Unfortunately, in March 2022, the patient developed symptoms such as vaginal bleeding, abdominal distension, and oliguria. After a repeat CT scan, a large mass in the pelvis was found again (**Figure 2D**). At the same time, multiple metastases appeared around the lungs and spine.

**Figure 3:**
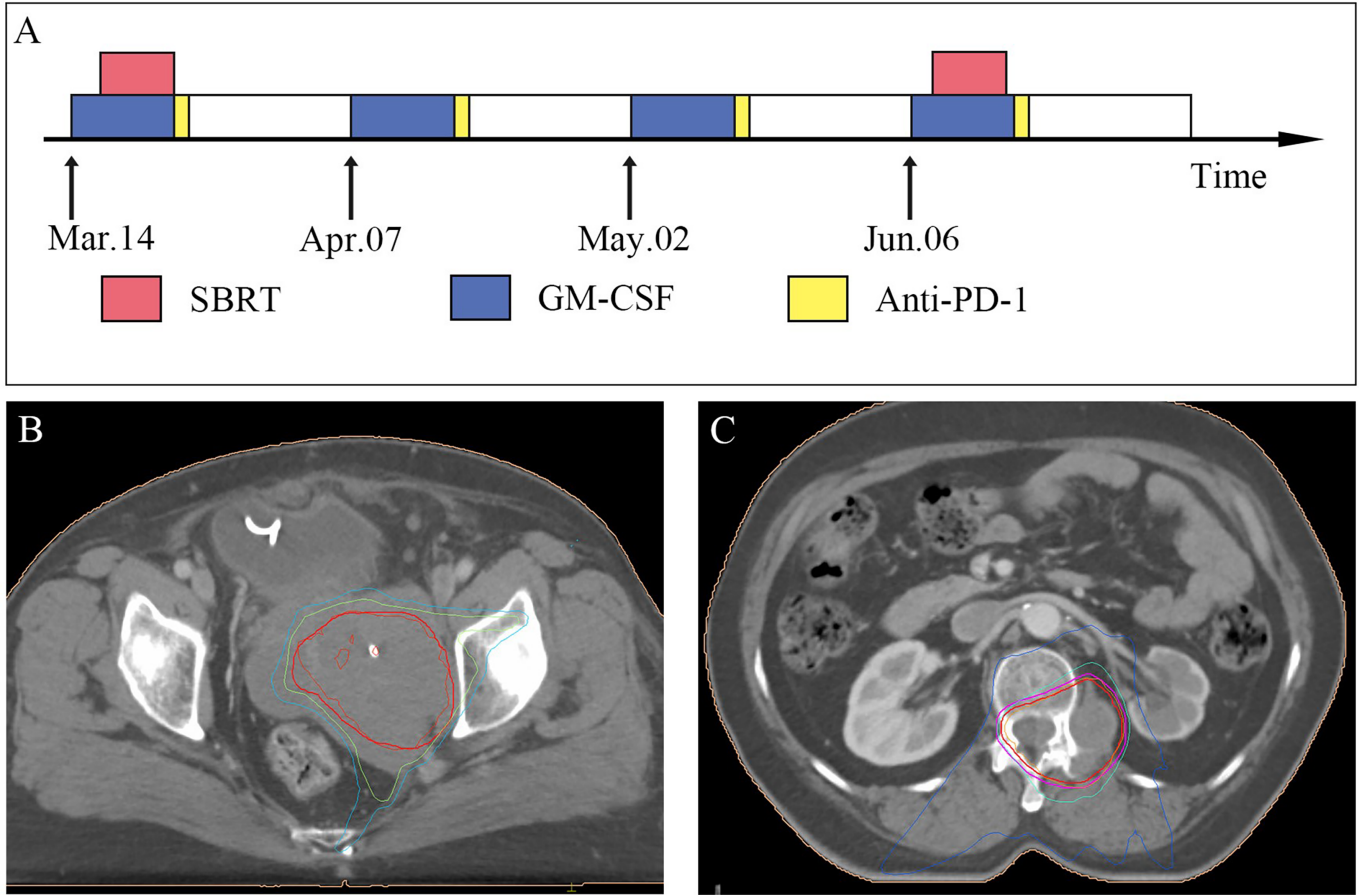
**(A)** The patient’s treatment schedule for 4 cycles. Subcutaneous injection of GM-CSF 200 mg was used on days 1-7. Immunotherapy with tislelizumab 200 mg on day 8. For the first and fourth time, SBRT was performed simultaneously with GM-CSF. **(B)** Radiotherapy treatment plan (isodose lines: 30 Gy, 24 Gy, 20 Gy in red, green, light blue respectively). **(C)** Radiotherapy treatment plan (isodose lines: 22.8 Gy, 20 Gy, 11.4 Gy in orange, light blue, dark blue respectively).

Treatment guidelines for PEComa were lacking and still are. However, the patient had severe local symptoms and positive tumour PD-L1 expression. After discussion with a multidisciplinary committee, we formulated a triple therapy regimen of anti-PD-1, GM-CSF, and SBRT for the patient (**Figure 3A**). Considering the large mass in the patient’s pelvis, to protect the surrounding organs as much as possible, the SBRT target volume was made slightly smaller than the actual tumour (**Figure 3B**). The patient started SBRT on March 14 at a dose of 30 Gy/6 Fx. GM-CSF was started at the same time as radiotherapy at 200 μg per day for 7 consecutive days. Tislelizumab (200 mg) was administered on Day 8. On April 7 and May 2, GM-CSF 200 μg daily was subcutaneously injected and continued for 7 days, and tislelizumab 200 mg was injected on the 8th day. Before the third use of tislelizumab, abdominal distension was significantly reduced, vaginal bleeding was completely controlled, and urine output had returned to normal. However, starting in May, the patient developed low back pain that gradually worsened. Combined with imaging, we believe that the pain is caused by a paraspinal tumour. We treated this tumour on June 6 with a triple therapy that included SBRT (**Figure 3A**). GM-CSF was administered continuously for 7 days at 200 μg daily. SBRT was started from Day 3 of GM-CSF use. The target area of SBRT included paraspinal tumour and tumour invading into the spinal cavity (**Figure 3C**). The radiation dose was 24 Gy/4 Fx. Tislelizumab (200 mg) was administered on Day 8. Shortly after the end of radiotherapy, the patient’s symptoms of low back pain were significantly relieved. In July, the patient developed oedema of the extremities, which was considered to be related to immune-related hypothyroidism. At the same time, the patient was reluctant to continue treatment due to economic reasons.

**Figure 4:**
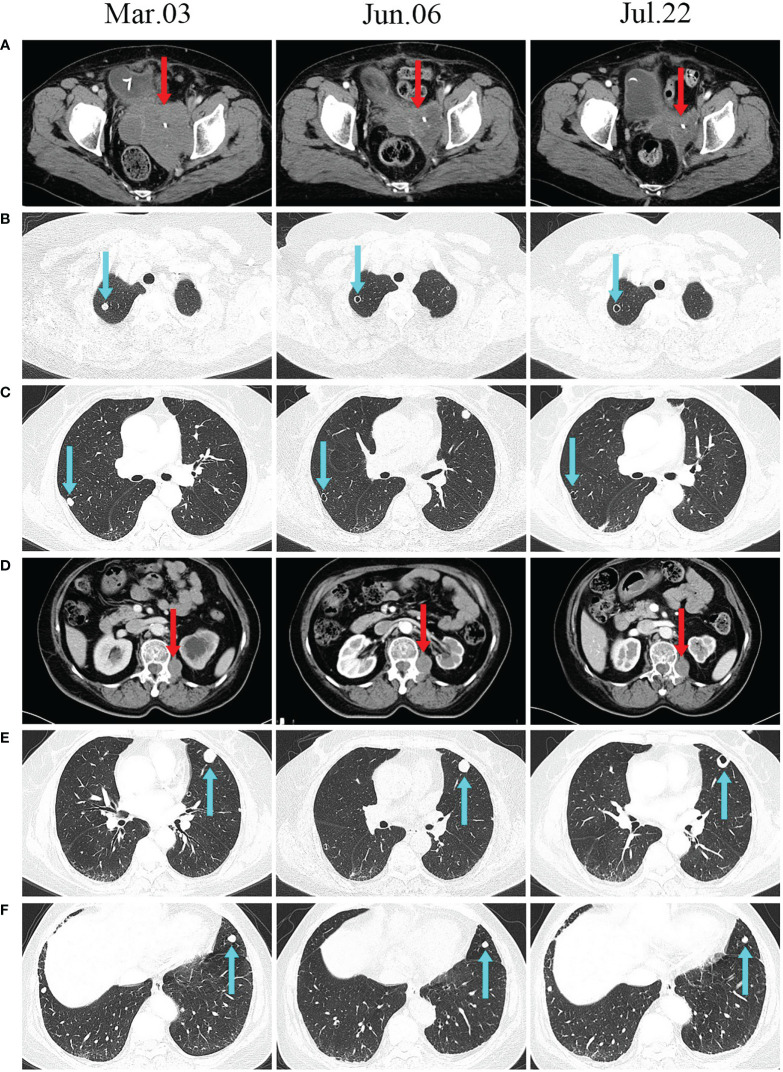
CT scans before and after treatment. **(A, D)** CT scans show changes in the size of irradiated lesions. **(B, C, E)** Unirradiated lung lesions developed cavities after treatment. (**F**) The unirradiated lesions in the lungs gradually decreased after treatment.

The patient’s systemic tumour was evaluated in June, and we found that the mass in the pelvis was significantly smaller than it had been in March (**Figure 4A**), and the two nonirradiated metastases in the lungs had transformed into thin-walled cavities (**Figures 4B, C**). There were no significant changes in other metastases. At the most recent imaging evaluation (July 22), the patient’s lumbar paravertebral tumour had not shrunk significantly (**Figure 4D**). However, we were pleasantly surprised to find a cavity in one of the larger metastases in the left lung (**Figure 4E**). Another pulmonary metastasis had shrunk by 30% in its largest diameter since treatment in March (**Figure 4F**). The patient is currently in a stable condition and is being treated with thyroid hormone replacement.

During treatment, the main adverse reactions were fever, myalgia, generalized oedema and immune-related hypothyroidism. According to the Common Terminology Criteria for Adverse Events (version 5.0), fever and myalgia were grade 1 adverse reactions. Fever and myalgia were believed to originate from the use of GM-CSF. After subcutaneous injection of GM-CSF, oral ibuprofen sustained-release capsules were helpful in significantly relieving fever and myalgia. Generalized oedema and immune-related hypothyroidism were grade 2 adverse reactions. The patient’s thyroid function was checked in June; thyroid stimulating hormone (TSH) was found to be slightly elevated, and serum free triiodothyronine (fT3) and free thyroxine (fT4) were found to be reduced. We considered that this could have been an immune-related adverse event. However, the patient did not have symptoms such as fatigue, chills or oedema. Therefore, we continued to use triple therapy, including PD-1 inhibitors. However, in July, the patient developed generalized oedema, and TSH increased more than in June. Although there were no symptoms of fatigue or chills, the patient was still given thyroxine replacement therapy. Afterwards, the patient no longer had systemic oedema, and TSH returned to normal.

## Discussion

Due to low incidence, there are few systematic studies of PEComa. A patient with advanced malignant PEComa who achieved long-term survival with nivolumab was reported by Michael Lattanzi et al. (5). It should be noted that the PD-L1 expression in this patient’s tumour exceeded 50%. This may be associated with a good prognosis. A high mitotic index and rich vascularization are features of malignant PEComa that may support radiation therapy as a reasonable treatment. In a patient with liver PEComa, the tumour shrank significantly, and complete resection was achieved after receiving SBRT (6). The role of PD-1 inhibitors and radiation therapy in the treatment regimen of PEComa deserves further investigation.

Recently, triple therapy with the PD-1 inhibitor GM-CSF and SBRT has been shown to be safe and may be used as a salvage therapy for refractory tumours (14, 16). In this case, we used the triple combination of a PD-1 inhibitor, GM-CSF and SBRT to treat PEComa and obtained a good curative effect. This therapeutic combination was reported for the first time in the treatment of PEComa. SBRT rapidly relieved this patient’s local symptoms. Her lung metastases showed abscopal effects. Radiation therapy has been shown to enhance antitumor immune responses and synergize with PD-1 inhibitors (7). Studies have shown that the combined use of radiotherapy and GM-CSF can help to produce abscopal effects (17). Radiation therapy can turn tumours into *in situ* vaccines by releasing large amounts of TAA (18). GM-CSF acts as an immune adjuvant to activate and promote dendritic cell (DC) maturation (19). As the most important antigen-presenting cells, mature DCs can activate naive CD4+ T cells and CD8+ T cells (20). Ultimately, the antitumor effect of the immune system is enhanced. In addition, radiotherapy also activates the cGAS-STING pathway, which increases tumour PD-L1 expression (21). On this basis, the use of immune checkpoint inhibitors helps to further improve the antitumor effect. One study recommends multisite radiotherapy instead of single-site irradiation, which can expose as much TAA as possible (13). In this case, the metastases in **Figure 4E** did not change significantly after the first irradiation. However, after the second irradiation of the paravertebral metastases, obvious cavitation appeared. This may be related to tumour heterogeneity among different metastases. In conclusion, the combined use of anti-PD-1, GM-CSF and multisite SBRT may be beneficial for the treatment of advanced malignant tumours with multiple metastases and is worthy of further study.

Taken together, triple therapy is effective in PD-L1-positive advanced malignant PEComa. The underlying mechanism may involve multiple aspects, such as exposure to tumour-associated antigens and changes in the tumour microenvironment. The exploration of malignant PEComa in immunotherapy and radiotherapy deserves further study.

## Data availability statement

The original contributions presented in the study are included in the article/supplementary material. Further inquiries can be directed to the corresponding authors.

## Ethics statement

The studies involving human participants were reviewed and approved by Ethics Committee of The Affiliated Yixing Clinical School of Medical School of Yangzhou University. The patients/participants provided their written informed consent to participate in this study. Written informed consent was obtained from the participant/patient(s) for the publication of this case report.

## Author contributions

All authors listed have made a substantial, direct and intellectual contribution to the work, and approved it for publication.
